# Using Flame-Assisted Printing to Fabricate Large Nanostructured Oxide Thin Film for Electrochromic Applications

**DOI:** 10.1186/s11671-020-03450-6

**Published:** 2020-11-23

**Authors:** Hualin Fan, Wei Yan, Yicheng Ding, Zhihao Bao

**Affiliations:** Shanghai Key Laboratory of Special Artificial Microstructure Materials and Technology, School of Physics Science and Engineering, Tongji University, Shanghai, 200092 China

**Keywords:** Flame-assisted printing, Nanostructure, Bismuth oxide, Thin films, Electrochromic

## Abstract

Flame spray pyrolysis was a process to produce oxide nanoparticles in a self-sustaining flame. When the produced nanoparticles were deposited on a substrate, nanostructured oxide thin films could be obtained. However, the size of the thin film was usually limited by the fixed substrate. Here, we demonstrated that thin film with a large area could be deposited by using the moving substrate, which was precisely controlled by servo motors. As a result, the flame tip could scan over the substrate and deposit the nanoparticles on it line by line, analogues to a printing process called flame-assisted printing (FAP). As an example, nanostructured bismuth-oxide thin films with a size of up to 20 cm × 20 cm were deposited with the FAP process. The bismuth-oxide thin film exhibited a stable electrochromic property with a high modulation of 70.5%. The excellent performance could be ascribed to its porous nanostructure formed in the FAP process. The process can be extended to deposit other various oxides (e.g., tungsten-oxide) thin films with a large size for versatile applications.

## Introduction

FSP was a process, in which solvent with the dissolved metal precursor was sprayed into liquid droplets. Then, the droplets combust into powders in a self-sustaining flame assisted with oxygen [1–5]. The size of the produced powders ranges from a few nanometers to micrometers. FSP can be easily employed to produce oxide nanopowders (e.g., SiO_2_, TiO_2_, CeO_2_ and Al_2_O_3_) in a commercial scale [6–9]. Although the nanopowders from the FSP process can be either dropped or cast on the substrate to form the thin films, those films are usually dense with a low surface area, lacking nanofeatures. For the applications such as sensors, electrochemical and photoelectrochemical (PEC) devices, thin films with the porous structure are preferred [10–12]. In the above applications, the porous structure can enhance the utilization of active materials, enlarge the contact area between the electrolytes and reactants, and alleviate the stress during the lithiation. Thus, their performance can be improved. For example, LiMn_2_O_4_ films were formed by flame spray deposition and in situ annealing method [13]. The highly porous thin film exhibited excellent cyclability. Kun et al. synthesized Li_4_Ti_5_O_12_ thin film for the high-performance and flexible all-solid-state battery [14]. Tricoli et al*.* [15] extended the use of FSP for the fabrication of EC/PEC water-splitting WO_3_ and BiVO_4_ electrodes. They found that the performance of direct FSP-made photoelectrodes had been greatly improved compared with those films cast with nanopowders from the FSP process. These pioneering works enabled the use of FSP as a powerful tool to directly and rapidly fabricate functional films with good performance. However, the size of the thin film was limited by the unmoved substrate. Bismuth oxide was one of the most fascinating electrochromic materials due to its high theoretical coloration modulation and environmental friendliness [16–27]. For example, bismuth oxide thin films prepared by sputtering or vacuum evaporation were found to be new electrochromic materials [16, 24]. Furthermore, bismuth oxide thin films from the sol–gel processes showed steady electrochromic efficiency [17]. However, their electrochromic performance should be further improved for the practical applications. In this study, we proposed a flame-assisted printing (FAP) process based on FSP to fabricate porous bismuth-oxide thin film on fluorine-doped tin oxide (FTO). The size of the thin film could reach 20 cm × 20 cm. The bismuth-oxide thin film deposited in this study exhibited excellent electrochemical properties with a coloration modulation of 70.5%. The excellent performance can be ascribed to the porous structure of thin films.

## Experimental

### Preparation of Bismuth-Oxide Thin Films

**Fig. 1:**
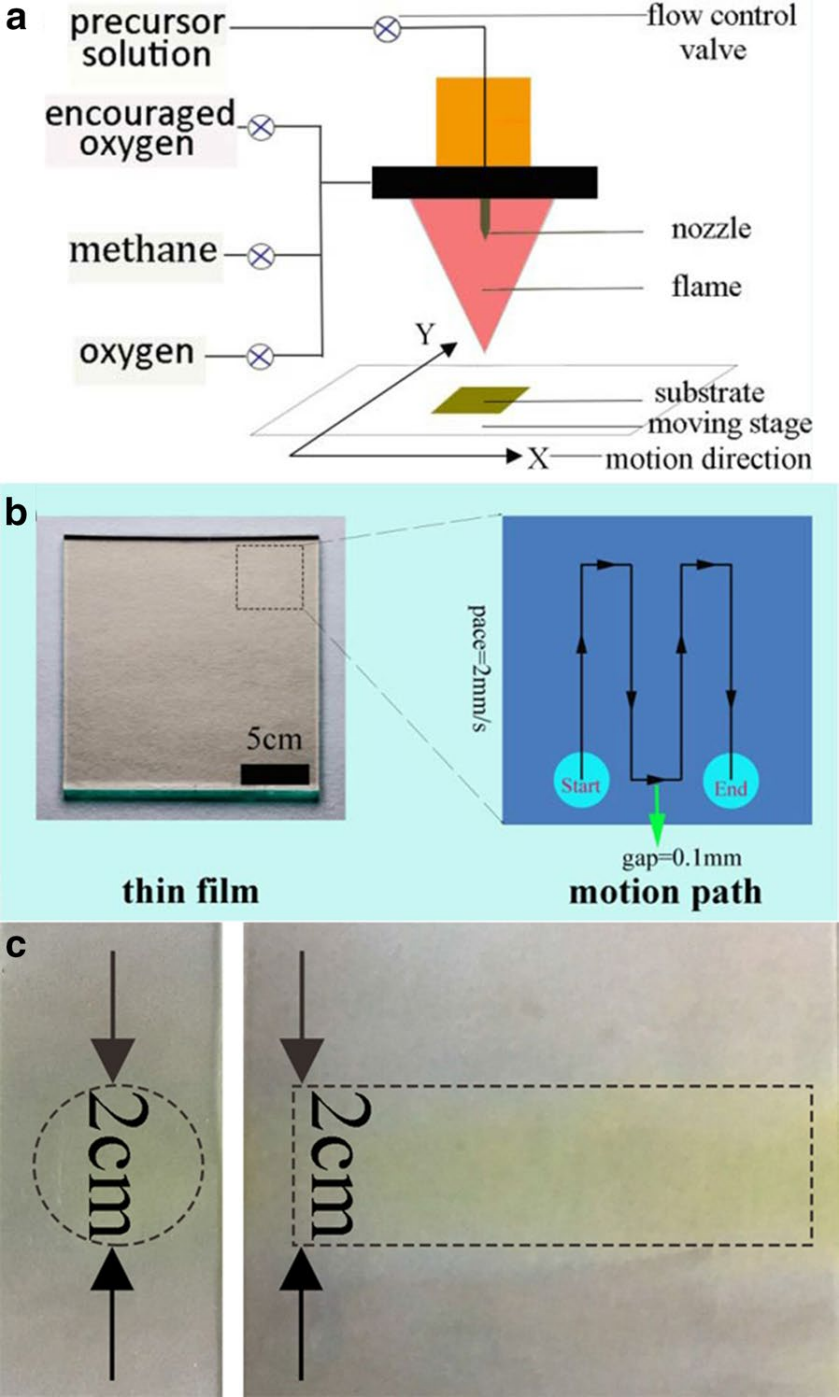
Schematic of the flame-assisted printing device (**a**) and process (**b**) to deposit large thin film on the substrate. **c** Optical images of the deposited spot and line by the FAP process

An FAP equipment was used for the direct deposition of bismuth-oxide thin films on FTO substrates (Fig. 1a). The thin films were prepared with following steps: before the bismuth-oxide thin films were prepared by the FAP process, 20 cm × 20 cm transparent conductive FTO glass substrates which have a sheet resistance of 10 Ω/sq were cleaned by ultrasonic with acetone, deionized water, ethanol and deionized water successively. The bismuth-oxide precursor was made by heating the mixture of bismuth carbonate (1.45 g), 2-ethylhexanoic acid (20 g) and deionized water (40 μL) to 160 °C under mechanical stirring. Precursor solutions with a total Bi atom concentrations of 1 and 5 mM were prepared by dissolving bismuth-oxide precursor in 2-2-4-trimethylpentane (15 ml). This solution was supplied with a syringe pump at a rate of 2 mL/min and dispersed into a fine spray with 2.541 L/min oxygen and pressure from 0.21 to 0.33 MPa was maintained across the nozzle tip during synthesis. The spray was ignited by a supporting flame made by 0.4 L/min of high-purity methane (99.9%) and 0.4 L/min of oxygen. A substrate holder placed at a certain distance below the burner was utilized for the deposition of bismuth-oxide thin films on FTO substrates with a suitable deposition temperature of 500 °C. The FAP process continued for 6 min. More specifically, films were deposited on the FTO substrates, which were placed on a moving stage. In the end, thin-film samples were annealed at a rate of 5 °C/min from room temperature up to 550 °C in air for 2 h.

### Preparation of Tungsten-Oxide Thin Films

The precursor solution was prepared by adding tungsten hexachloride (29.742 mg) to anhydrous ethanol (30 ml). The formed mixture was stirred at room temperature for 1 h. The substrate temperature was controlled in the range of 400–500 °C during the deposition.

### Characterization Methods

The phase composition of the bismuth-oxide thin films was analyzed by X-ray diffraction (Bruker XRD, D8 Advance) using CuKα radiation (40 kV/30 mA). The surface morphologies of the thin films were investigated by field-emission scanning electron microscope (FESEM) with an Ultra 55 operating at 10 keV. Roughness measurements were taken using a profilometer (Bruker Contour, GT K 3D) with a cut-off value of 0.23 mm. Three different regions were evaluated in specimens to determine the roughness Ra. The EC behavior of bismuth-oxide thin films was evaluated with a standard three-electrode electrochemical cell using AutoLab 302N electrochemical workstation. The bismuth-oxide thin-film-coated FTO glass was applied as a working electrode, platinum sheet and Ag/AgCl electrode were served as a counter electrode and a reference electrode, respectively. 1 M solution of LiClO_4_ dissolved in propylene carbonate (PC) was used as the electrolyte. Cyclic voltammetry (CV) was carried out with the scan rate of 2 mV/s or 5 mV/s and voltage range of − 2 to 2 V and − 1 to 1 V for bismuth-oxide thin films and tungsten-oxide thin films, respectively. The optical transmittance change of the bismuth-oxide and tungsten-oxide thin films during the charging and discharging process in CV cycling was in situ recorded by a miniature spectrometer (Ocean optics, FLMT01617) at a fixed wavelength of *λ* = 550 nm.

## Results and Discussion

**Fig. 2:**
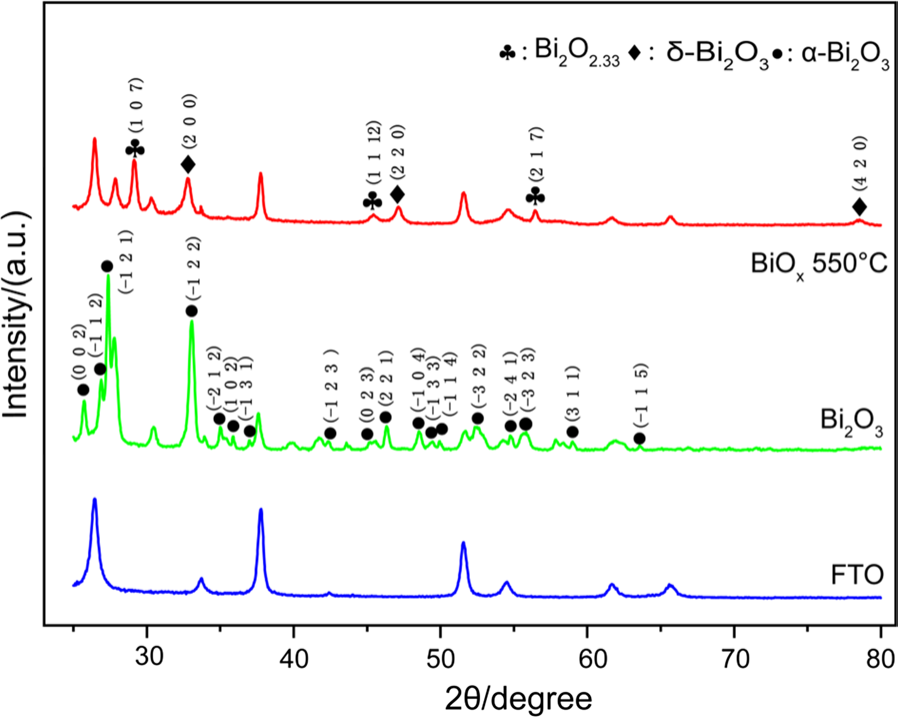
X-ray diffraction patterns of FTO substrate (bottom), bismuth-oxide thin film (middle) as deposited, and the thin film (top) after the heat treatment at 550 °C

**Fig. 3:**
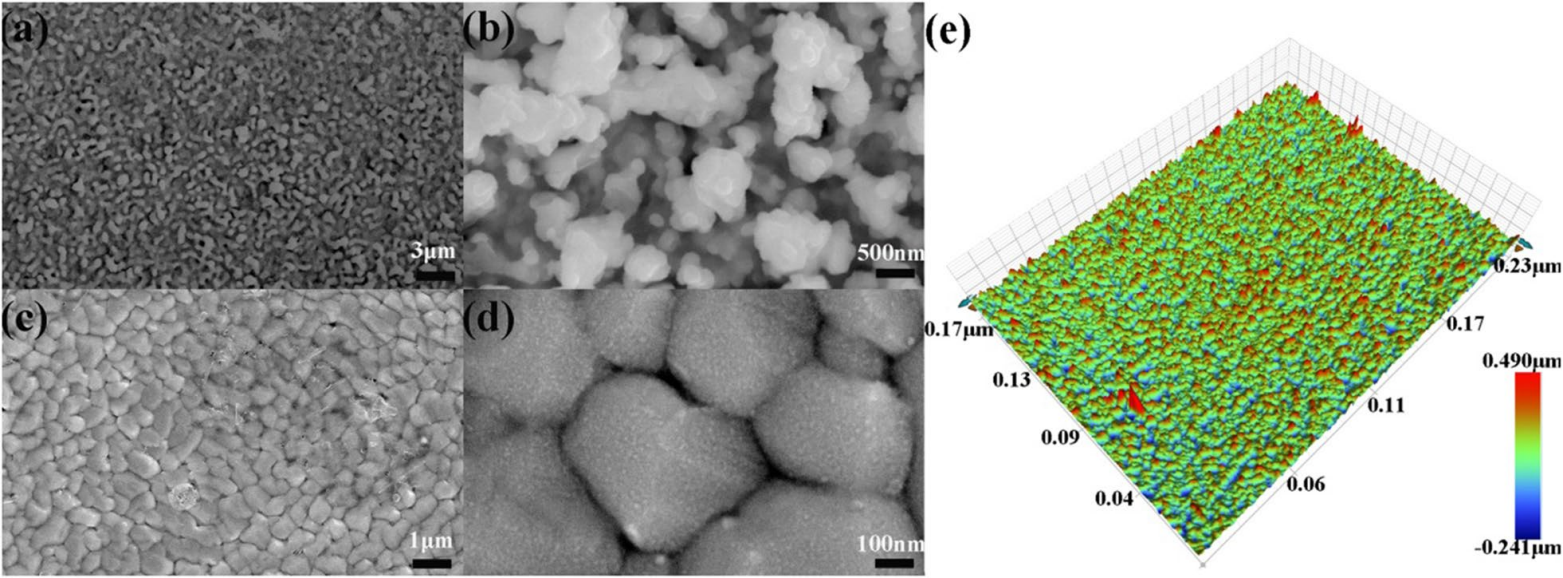
Morphology characterization of the bismuth-oxide thin films. SEM images (**a**, **b**) of bismuth-oxide thin film on the FTO substrates from 5 mM solution. SEM images (**c**, **d**) of the bismuth-oxide thin film from 1 mM solution with heat treatment at 550 °C. And **e** roughness profile of bismuth-oxide thin film from 5 mM solution

**Fig. 4:**
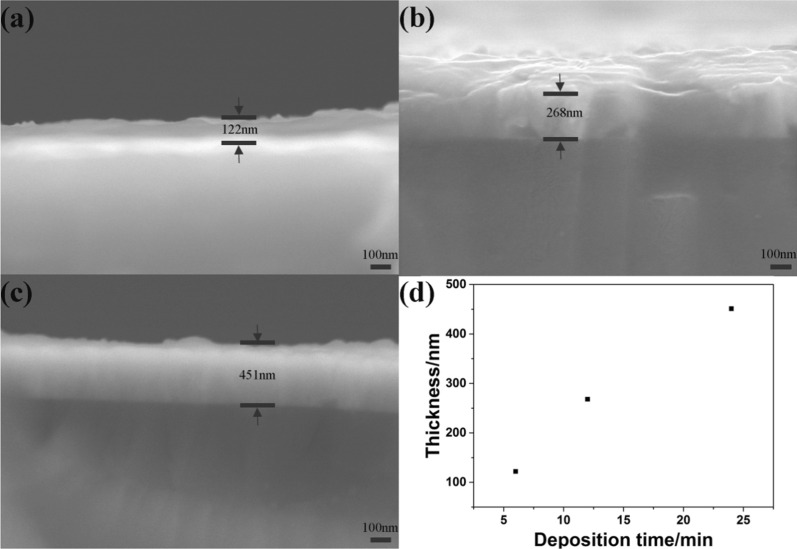
SEM images of cross sections of the thin films corresponding to the deposition times of **a** 6 min, **b** 12 min and **c** 24 min, and **d** the relationship between the thickness of the thin films versus the deposition times

The FAP device and process are illustrated in Fig. 1a, b. During the process of FAP, porous structure comes into being with the formation of nanoparticles, primary particles and large particles [28]. The growth of nanoparticles and primary particles gives multilevel structure, while primary particle collision and large particle separation generate the porous structure [29]. There are a lot of factors such as precursor concentration that have an impact on the final particle morphology, structure and performance. To deposit bismuth-oxide thin films, precursor solutions with concentrations of 1 and 5 mM were pumped with a speed of 2 ml/min into the nozzle and sprayed it into the droplets. They were then combusted into the oxide nanoclusters. The formed nanoclusters collided to form nanoparticles and deposited on the FTO substrate, which located at the moving stage. It was precisely controlled by the servo motors. As a result, the flame swept the substrate with a speed of 2 mm/s line by line. The gap between the neighboring lines was controlled to be 0.1 mm as indicated in Fig. 1b. The thickness of the film was controlled by the precursor concentration and repeating time. This line-by-line depositing process was analogous to a paper-printing process. Thus, we called this process as flame-assisted printing. FAP process was also used to deposit a spot when no scanning was performed and a line with the scanning process from 5 mM bismuth oxide precursor. The optical images of the deposited spot and line are shown in Fig. 1c. The size of the spot and the width of line were ~ 2 cm. Thus, FAP process may able to print an arbitrary 2D shape, though the resolution of the shape is limited to 2 cm. After the deposition, the obtained thin film was annealed at 550 °C for 2 h. X-ray diffraction (XRD) patterns in Fig. 2 revealed their crystalline and phase structures. Before the annealing, there was only α-phase (JCPD card No. 71-0465) for Bi_2_O_3_. While after 550 °C annealing, there were two new phases. They were δ-Bi_2_O_3_ phase (JCPD card No. 76-2478) and non-stoichiometric Bi_2_O_2.33_ phase (JCPD card No. 27-0051). The latter one was related to the oxygen vacancy due to annealing in the stagnant air [30]. The above phase transition was consistent with the previous studies [17]. The morphology of obtained bismuth-oxide thin film from 5 mM precursor was examined with a scanning electron microscopy (SEM). As shown in Fig. 3a, b, the whole structure exhibited a macroporous structure with secondary particles of several hundred nanometers. As a comparison, a thin film was also deposited from 1 mM precursor. It turned to be a solid film with the secondary particles approaching 1 μm as shown in Fig. 3c, d. The difference in the concentrations of the precursor solution apparently caused the change of morphology. And according to the large-scale characterization by using a profilometer, the mean Ra value of the Bi_2_O_3_ thin film from 5 mM precursor was measured to be 29 ± 2 nm (Fig. 3e), which was consistent with SEM analyses. Under high concentration, particles easily collided with each other and then branched secondary particles deposited on the substrates. While under low concentration, the primary particle was small enough to fill the gaps among the deposited particles. The morphology of the thin film appeared less porous. Thus, the morphology and pore structure of the thin film can be tubed with the concentration of the precursor in the FAP process. In addition, the further research on the influence of deposition time on film thickness was carried out. SEM images of the cross sections of the films for deposition times of 6 min, 12 min and 24 min are shown in Fig. 4. Their thicknesses increased with the time. Thus, FAP process also could control the thicknesses of the film by varying the deposition time.

**Fig. 5:**
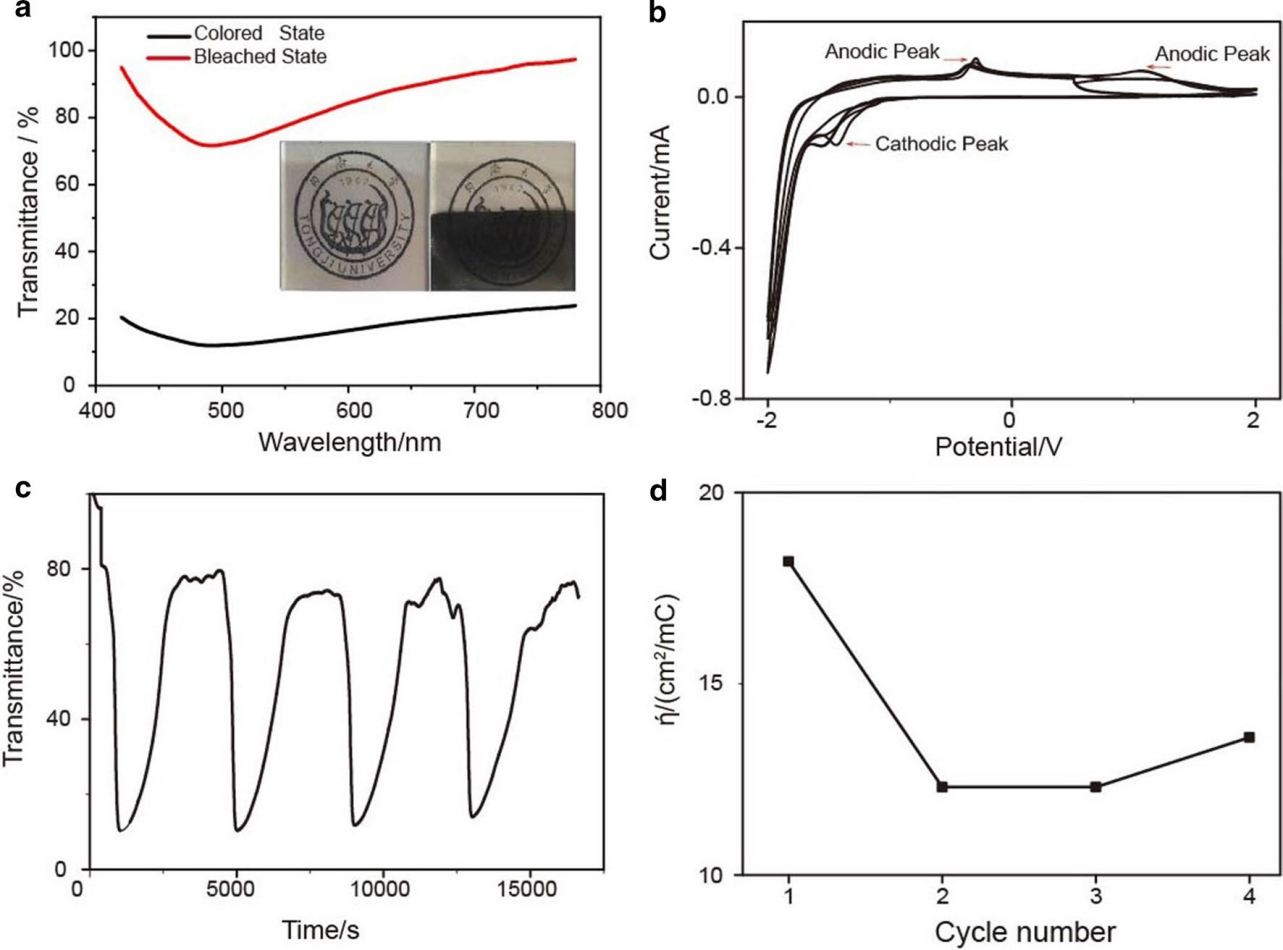
**a** Optical spectra in the bleached state and colored state, **b** cyclic voltammograms, **c** time-dependent luminous transmittance at 550 nm and **d** coloration efficiency of the bismuth-oxide thin film deposited with 5 mM solution

The electrochromic property of the bismuth-oxide thin film from the 5 mM precursor was further investigated. The color of the thin film changed from light yellow in the bleached state to black in the colored state as shown in its optical image (Fig. 5a, inset). The optical spectra further revealed that the transmission was in a range of 75–100% at the bleached state, while at the colored state, the transmission of the thin film was in a range of 10%–30%. The CV curve (Fig. 5b) indicated that there were a cathodic peak at − 1.3 V and two anodic peaks at 0.1 V and 1.2 V, which are typical for the lithium intercalation of lithium ions into the bismuth-oxide structure forming Li_*x*_Bi_2_O_3_ while charging followed by reversible deintercalation of Li_*x*_Bi_2_O_3_ back to Bi_2_O_3_ during discharging, due to the Bi_2_O_3_/Li_*x*_Bi_2_O_3_ redox reaction. It corresponded to the following reaction [16]:

1 $${\text{Bi}}_{{2}} {\text{O}}_{{3}} + x{\text{Li}}^{ + } + x{\text{e}}^{ - } \leftrightarrow {\text{Li}}_{x} {\text{Bi}}_{2} {\text{O}}_{3}$$

The stable redox couple reactions of bismuth oxide cause the electrode to perform with good reversibility and demonstrate good stability by hardly altering shape, size and position of its anodic and cathodic peaks. The sharp and well-defined peaks indicate fast ion de- and insertion. The CVs in Fig. 5b of bismuth-oxide thin-film electrodes show the characteristic behavior of two-step extraction (peaks at 0.1 V and 1.2 V) and insertion (peak at − 1.3 V) of the lithium ions in deposit with uniform grain size indicating a successful film formation and densification took place. All electrodes show uniformity in particle sizing after successful film preparation. It indicates that the quality of the surface after heat treatment allows homogeneous porous layer stacking. However, the long-term cyclic stability of this bismuth-oxide thin-film electrode is not as good as expected due to poor adhesion to the FTO substrate. We leave improving this cyclic stability by adjusting the precursor concentration and annealing temperature as our future work.

**Fig. 6:**
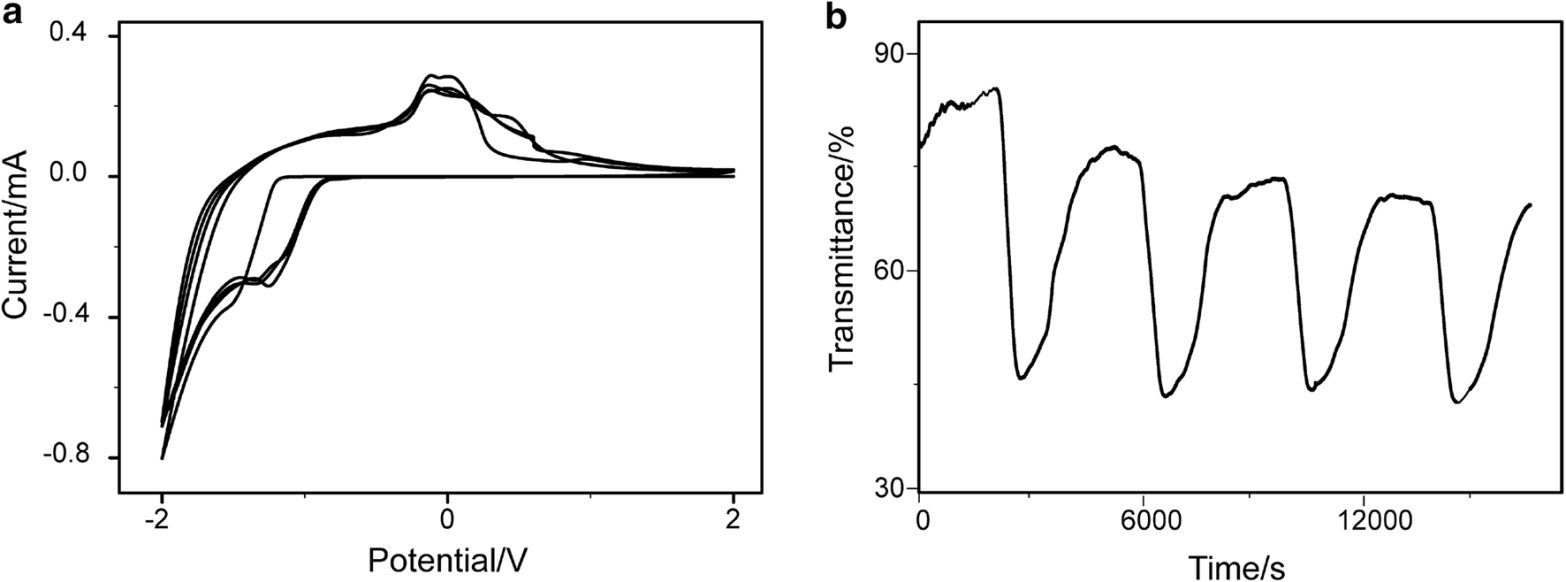
CV (**a**) and time-dependent transmittance (**b**) of bismuth-oxide thin film deposited from 1 mM solution after heat treatment at 550 °C

The time-dependent optical transmittance of the bismuth-oxide thin films was in situ recorded during the CV measurement, as shown in Fig. 5c. The transmittance wavelength was set at 550 nm, which was highly sensitive to human eyes [31]. After the first cycle, the maximum and minimum transmittances were 80.7% and 10.2%, respectively. Both transmittance values at the bleached state and colored state were kept stably. The minimum transmittance at the colored state and the maximum transmittance at the bleached state were recorded as *T*_c_ and *T*_b_, respectively. Then optical contrast Δ*T*_*λ*=550 nm_ was defined as Δ*T* = *T*_b_ − *T*_c_. It is obvious that the bismuth-oxide thin film had a large optical contrast beyond 70%. The thin films had a slight optical degradation during the 2nd, 3rd and 4th cycles. The difference between colored and bleached states can be seen. This is mainly caused by the degradation in the reaction [17, 32], and the porous structure leads to an incomplete reaction [33]. Here, EC response time, T_0.5_, is defined as the time at which the optical transmittance reaches 50% of the coloration/bleach state at the wavelength of 550 nm. As shown in Figs. 5c and 6b, *T*_0.5_ = 120 s for bismuth-oxide thin film deposited from 5 mM precursor solution and *T*_0.5_ = 300 s for bismuth-oxide thin film deposited from 1 mM precursor solution, respectively. The faster bleach rate could be ascribed to its porous nanostructure formed in the FAP process. Conventionally, coloration efficiency *η* (CE) is used to judge the EC performance with the following formula [17]:

2 $$\eta \left( {{\text{CE}}} \right) = \frac{{\Delta {\text{OD}}}}{Q} = \frac{{\log \left( {T_{{\text{b}}} /T_{{\text{c}}} } \right)}}{Q}$$

where *T*_b_ and *T*_c_ are the bleached and colored transmittance values at a given wavelength as mentioned above, Δ*OD* is the difference of optical density and *Q* is the corresponding inserted/extracted charge density. In this work, charge densities are calculated from the CV curves. The CEs of bismuth-oxide thin film were all larger than 10.0 cm^2^/C as shown in Fig. 5d. The CEs of bismuth-oxide thin film from 5 mM precursor were nearly the same as ones from the sol–gel derived thin film [17] and much higher than that of 3.7 cm^2^/C reported in ref [16]. As for the bismuth-oxide thin film deposited from 1 mM precursor solution, the anodic and cathodic peaks were broadened, as shown in Fig. 6a. A significant peak broaden in anodic coloring was usually observed when the precursor solution concentration was far below 5 mM. This can be explained by the lower concentration which caused less solid/solid interfaces to the composite film due to the less porous structure [34]. Meanwhile, the optical contrast was only 30–40% (Fig. 6b), and CE was 2.7–3.4 cm^2^/C. Compared with the thin film from 5 mM precursor solution, the poorer performance could be ascribed to the relatively solid nature of the thin film. In the electrochemical reaction, the electrolyte had less contact area with the active material. Thus, less active material participated in the reaction. Moreover, the degradation was obvious during the subsequent cycle due to the structure’s instability, which was caused by the change of the volume of the thin film in the reaction, while porous structure in the thin film from 5 mM solution could accommodate such change.

**Fig. 7:**
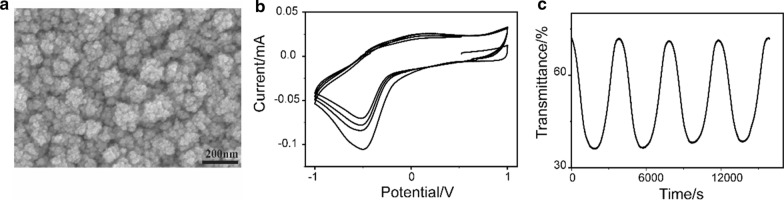
**a** SEM image, **b** cyclic voltammogram and **c** time-dependent luminous transmittance of the tungsten-oxide thin film at 550 nm

When the FAP equipment is extended to the synthesis of other oxides such as tungsten oxide as mentioned, the SEM image (Fig. 7a) exhibited the microstructure of the deposited tungsten-oxide thin film, while the precursor concentration is 2.5 mM. It had secondary particles, which aggregated a lot of spherical primary particles, which forms the cauliflower structure. More specifically, it appears that secondary particles are larger and less porous than the bismuth-oxide thin film from 5 mM precursor. There are anodic and cathodic peaks at 0.1 and − 0.5 V, which is typical for the lithium deintercalation and intercalation (Fig. 7b). Coloration/bleach in WO_3_ thin films is due to the insertion and extraction of lithium ion, following the reaction below [35]:

3 $${\text{WO}}_{3} + x{\text{Li}}^{ + } + x{\text{e}}^{ - } \leftrightarrow {\text{Li}}_{x} {\text{W}}^{6 + }_{(1 - x)} {\text{W}}^{5 + }_{x} \,{\text{O}}_{{3}}$$

In WO_3_ thin film, the inserted electrons reduce some W^6+^ ions to W^5+^ and polarize their surrounding lattice to form small polarons which cause the optical absorption. In bismuth-oxide thin film, the coloration/bleach may follow the same mechanism. However, the unstable chromatic properties may be caused by the stress build-up due to volume change or dissolution of Li_*x*_Bi_2_O_3_ in the electrolyte during the reaction. The detachment of bismuth-oxide thin film from FTO substrates was often found after several coloration/bleach cycles. The bleached and colored states kept stable in 4 cycles (Fig. 7c). The chromatic property of tungsten oxide is more stable than bismuth oxide [16, 17, 35–39]. The optical contrast was around 35%. It was relatively low, but not lower than bismuth-oxide thin film deposited from 1 mM precursor. To get a higher value, the thickness of tungsten-oxide thin film should be increased. The first coloration efficiency of tungsten oxide was 3.4 cm^2^/C, which was nearly the same as the data reported [16] and between the bismuth-oxide thin films from 1 and 5 mM precursor. Magnetic sputtering, pulsed laser deposition and chemical vapor deposition are several popular coating methods. They can be used to produce high-quality thin film with a thickness precision of several nanometers. However, all of them are difficult to produce large-area thin films due to the size limitation of deposition chamber. While FAP process can easily prepare large samples, it can be operated in the open atmosphere. Meanwhile, though the process cannot control the thickness very precisely, it can adjust the morphology of the thin film for the specific applications. The result above indicated that FAP could tune the nanostructure of the deposited film by precursor concentration, which further influenced its electrochromic performance.

## Conclusion and Outlook

Large-area bismuth-oxide thin films were successfully prepared by a FAP process. The morphology of the thin films could be tuned with the deposition parameters such as the concentration of the precursor solution. The bismuth oxide with porous nanostructure exhibited excellent electrochromic property, with a maximum optical contrast of 70.5% and a coloration efficiency above 10.0 cm^2^/C. This FAP process can be extended to the synthesis of other porous nanostructured materials for applications in energy storage and conversion.

## Data Availability

All data generated or analyzed during this study are included in this published article.

## Abbreviations

FSP: Flame spray pyrolysis
FAP: Flame-assisted printing
EC: Electrochromic
PEC: Photoelectrochemical
FTO: Fluorine-doped tin oxide
FESEM: Field-emission scanning electron microscope
PC: Propylene carbonate
XRD: X-ray diffraction
SEM: Scanning electron microscopy
CE: Coloration efficiency
